# Multifractal analysis of cellular ATR-FTIR spectrum as a method for identifying and quantifying cancer cell metastatic levels

**DOI:** 10.1038/s41598-023-46014-1

**Published:** 2023-11-02

**Authors:** Ayan Barbora, Sirish Karri, Michael A. Firer, Refael Minnes

**Affiliations:** 1 Department of Physics, Ariel University, 40700 Ariel, Israel; 2 Department of Chemical Engineering, Ariel University, 40700 Ariel, Israel; 3 Adelson School of Medicine, Ariel University, 40700 Ariel, Israel; 4 Ariel Center for Applied Cancer Research, Ariel University, 40700 Ariel, Israel

**Keywords:** Cancer screening, Biological physics, Metastasis, Colon cancer, Melanoma

## Abstract

Cancer is a leading cause of mortality today. Sooner a cancer is detected, the more effective is the treatment. Histopathological diagnosis continues to be the gold standard worldwide for cancer diagnosis, but the methods used are invasive, time-consuming, insensitive, and still rely to some degree on the subjective judgment of pathologists. Recent research demonstrated that Attenuated Total Reflection-Fourier Transform Infrared (ATR-FTIR) spectroscopy can be used to determine the metastatic potential of cancer cells by evaluating their membrane hydration. In the current study, we demonstrate that the conversion of ATR-FTIR spectra using multifractal transformation generates a unique number for each cell line’s metastatic potential. Applying this technique to murine and human cancer cells revealed a correlation between the metastatic capacity of cancer cells within the same lineage and higher multifractal value. The multifractal spectrum value was found to be independent of the cell concentration used in the assay and unique to the tested lineage. Healthy cells exhibited a smaller multifractal spectrum value than cancer cells. Further, the technique demonstrated the ability to detect cancer progression by being sensitive to the proportional change between healthy and cancerous cells in the sample. This enables precise determination of cancer metastasis and disease progression independent of cell concentration by comparing the measured spectroscopy derived multifractal spectrum value. This quick and simple technique devoid of observer bias can transform cancer diagnosis to a great extent improving public health prognosis worldwide.

## Introduction

Cancer continues to be a major contributor to deaths worldwide^1, 2^. Timely detection of the disease and identification of at-risk individuals is crucial to success of effective treatments, which can greatly increase the survival rate of patients. The current technologies for screening and diagnosis involving imaging techniques are generally insensitive and detect disease only when tumor mass is already generated and visible^3^, Another concerning aspect of current cancer diagnosis is the variability of disease classification amongst pathologists. For example, a study in 2017 on inter-observer and intra-observer variability among pathologists using visual inspection of tissue sections observed that 17% of melanocytic lesions diagnosed in the US are incorrect^4^. Although diagnosticians utilize specific features from biopsy materials on microscopic slides, radiographs, and patients’ physical examination, the diagnoses incorporate individual perspectives in the processing, grading and categorization of medical information^4, 5^. As such, the reliability and predictive values of these diagnostic criteria have never been established with rigorous standards until now due to high levels of diagnostic discordance across pathologists in interpreting cancerous lesions^6–9^, demonstrating the serious state of both overdiagnosis and underdiagnosis^5, 10^ in current medical practice. Records show that pathologists’ interpretations of the same case on two occasions lacked reproducibility^4^, making this low level of diagnostic precision a serious clinical concern. Also, computer aided detection (CAD) tools that are widely used in clinical practice to aid the interpretation and diagnosis have been found to cause potential harms, such as higher recall and biopsy rates for screening mammography^11^. Thus, reliable and objective techniques are needed to validate and support pathologists’ visual assessments of cancerous lesions, which can overcome the subjective bias in observer-derived opinions about diagnostic certainty, perceived risk for disease progression, and suggested management.

Studies have previously reported that the degree of metastatic motility of cancer cells is related to their degree of cell membrane fluidity^12–17^. This higher fluidity of the plasma membrane derives from higher levels of hydration^18–20^. We previously demonstrated a dynamic Attenuated Total Reflection-Fourier Transform Infrared (ATR-FTIR) spectroscopy method sensitive for detecting the hydration levels of the plasma membrane^21^ which enables distinguishing cancer cells at different metastatic potentials by measuring the mid-IR spectra of the cells. We identified two spectral zones involving the absorption peaks of membrane protein and the ratio of structural water to non-structural water^21^. A method that converts these unique FTIR spectral curves, which incorporates absorption data over a range of wavelengths, into a characteristic parameter specific to the metastatic potential of the cell sample under assay can provide an effective method for detecting and identifying cancer.

The biological significance of these spectroscopic developments can be enhanced by coupling them to digital image analysis of changes in cellular features. Fractal dimension measures the space filled by an object, which is otherwise impossible to calculate using Euclidian geometry^22^. All natural structures exhibit irregularity and a property of self-similarity. The changes in these properties can be studied considering the changes in their fractal dimension. Multifractal analysis (MFA) is an extension to fractal dimension assay. MFA determines the changes in fractal structures, making it useful for analyzing signal patterns in biomedical imaging such as ECG (electrocardiogram, to analyze heart rate variability)^23^, EEG (electroencephalogram, to evaluate the brain’s electrical behavior)^24^ and X-ray images for characterization of soil macropore structures^25^. These recent developments show that MFA is a promising tool in the field of biology and medicine to statistically evaluate, extract, and represent chaotic graphical representations such as heartbeat, wave functions, mechanical vibrations, and possibly also FTIR spectra. The principle behind MFA is that different regions of a spectra have different fractal properties, providing information on a broad range of heterogenous phenomena.

Cancer cells membranes are more fluid than normal cells of the same genetic lineage^26^ with no significant locational differences in the stiffness of cells between the central and peripheral regions. These characteristic mechanical features arise from the modified hydration and molecular changes in the membranes of cancer cells with reference to healthy cells. These changes produce spectral alterations detectable by ATR-FTIR spectroscopy^21^. Here, we present a novel technique of MFA on FTIR spectra, enabling precise identification of cancer cells at different metastatic levels. The method was tested and verified on human colorectal cancer, murine and human melanoma cell lines. The experiments demonstrate a subjective-bias-free, spectrum-based statistical tool for cancer detection and disease progression.

## Results

### MFA of ATR-FTIR spectra of cells generate unique multifractal number corresponding to their levels of metastatic potential

MFA of spectra plots generated by the ATR-FTIR acquisitions were analyzed using BOX count method to determine the distribution of pixel values under multiple scaling values. To evaluate if the given spectra showed multifractal properties, we initially analyzed *f*_*(α)*_ versus *α* singularity spectra (see *Methods*). The resulting spectrum was found to show a typical concave curve which confirmed the input spectra was a perfect example of a multifractal structure.

**Figure 1:**
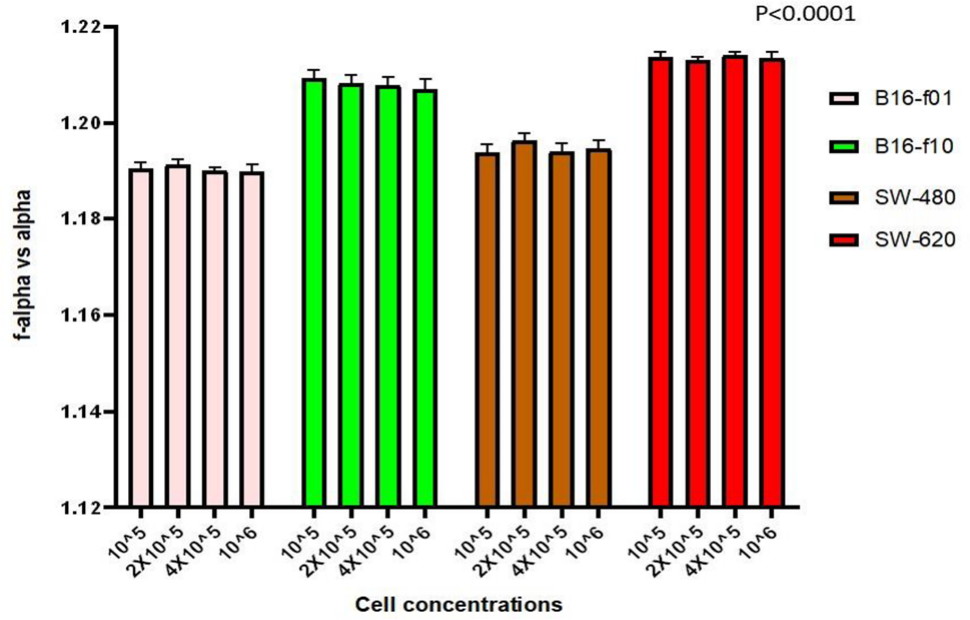
Multifractal number (*f*_*(α)*_ vs. *α*) of acquired ATR-FTIR Spectra from B16-f01, B16-f10, SW-480 and SW-620 cell lines over subsequently increasing concentration levels (10^5^, 2 × 10^5^, 4 × 10^5^ and 10^6^ cells/ml each). The values and error bars represent mean and standard deviation of the calculated multifractal number by MFA of 3 distinct ATR-FTIR spectra for each respective cell line and each respective concentration as indicated. Calculated *p* values are less than 0.0001.

B16-f01 and B16-f10 cells are subclones of different metastatic potential derived from the parental murine melanoma B16 line. They were isolated from lung cancer metastases cells formed following their injection into C57 mice: the B16-f10 cells are more aggressive than the B16-f01 cells^27^. MFA of acquired ATR-FTIR spectra of B16-f01 and B16-f10 cells at subsequently increasing concentration levels showed that the multifractal number from each cell type is independent of the concentration of cells used and unique to the cell line (Fig. 1). The higher metastatic potential cell line generates a higher multifractal number. The same trend was observed upon MFA of acquired ATR-FTIR spectra from the adenocarcinoma cell lines SW-480 and SW-620 (representing lower and higher tumorigenic and metastatic potential, respectively; see Methods) of human colorectal cancer disease over subsequently increasing concentration levels (Fig. 1); demonstrating a cell number independent higher multifractal number for higher metastatic potential.

The technique of ATR-FTIR spectra acquisition and multifractal number generation presented in this article requires the samples to be pressed using a screw top knob to maximize the contact of the cell membrane with the ATR diamond; stipulating that for a sample under analysis, the multifractal number generated as such would be independent of the cell concentration used. To test this hypothesis, samples of each cell type at subsequently increasing cell concentrations (10^5^, 2 × 10^5^, 4 × 10^5^ and 10^6^ cells/ml) were analyzed to generate their multifractal number from the acquired ATR-FTIR spectra (Fig. 1). The resulting observations indicate that higher and lower metastatic level (based on the physiological difference in membrane hydration levels detected by ATR-FTIR spectroscopy) of the same cell lineage under analysis can be classified by the characteristic multifractal number. Further, this assay was observed to be independent of the cell concentrations used for the same lineage under test.

### MFA identifies cancer cells with metastatic potential within a population of non-cancer cells

The ratio of metastatic cells to healthy cells in tissues increases as cancer disease progresses. Using MFA on acquired FTIR spectra, we tested whether our method could detect cancer cells of differing metastatic potential when they are mixed with increasing concentrations of non-cancerous cells. This experiment tests our technique’s ability to detect and objectively report disease progression from biopsy samples.

**Figure 2:**
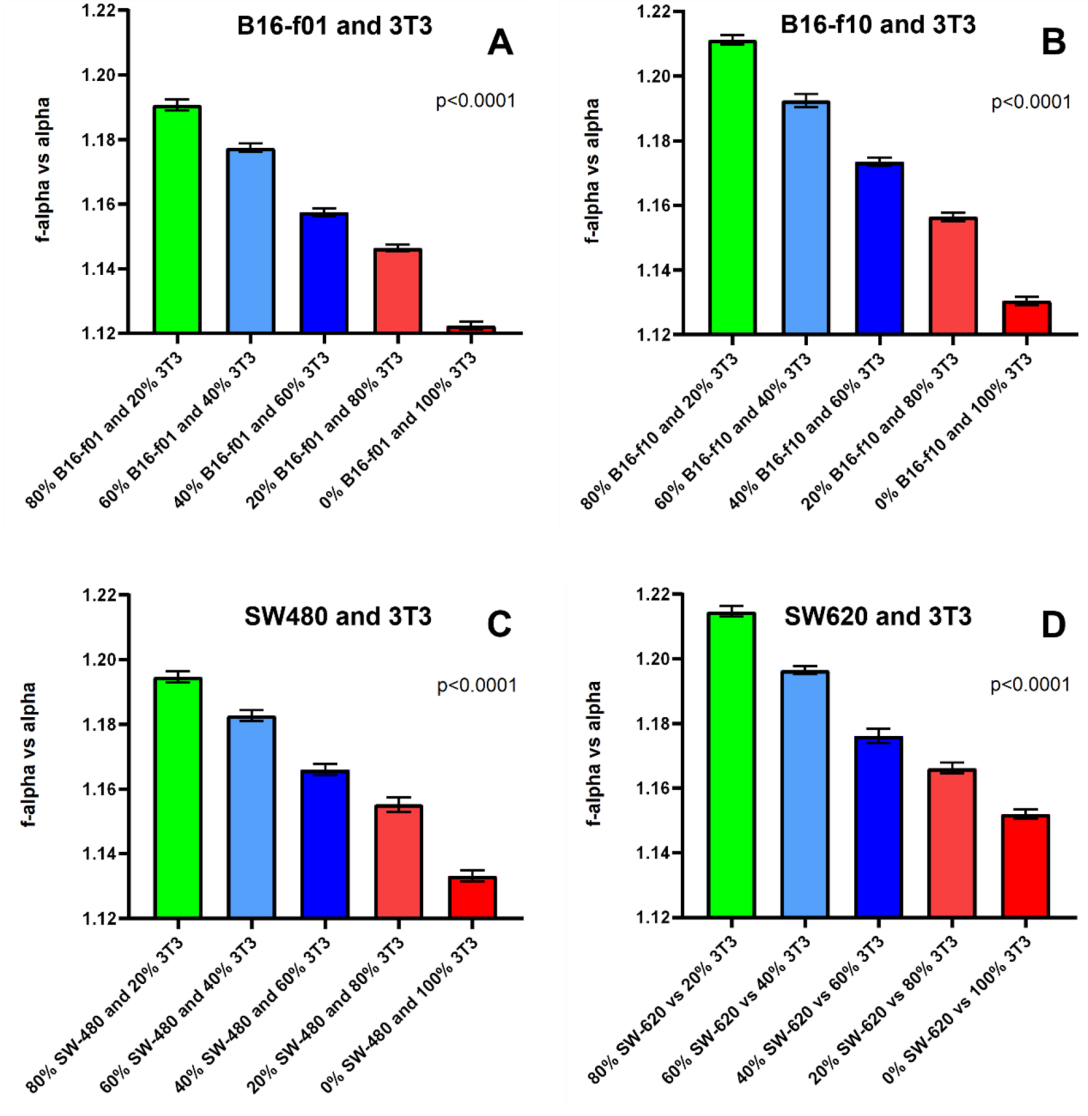
Multifractal number (*f*_*(α)*_ vs. *α*) of acquired ATR-FTIR Spectra from (**A**) B16-f01 mixed with 3T3 cells (**B**) B16-F10 mixed with 3T3 cells (**C**) SW-480 mixed with 3T3 cells and (**D**) SW-620 mixed with 3T3 cells; at the indicated percentages. Each data point represents a sample containing 10^6^ cells/ml in total. The values and error bars represent mean and standard deviation of the calculated multifractal number by MFA of 3 distinct ATR-FTIR spectra for each respective sample. Calculated *p* values are less than 0.0001.

We observed that as the ratio of cancer cells to non—cancerous 3T3 cells increases from 20 to 100% the associated multifractal number also increases in value (Fig. 2) for every cell type tested. This incremental cancer cell experiment is analogous to the *in-vivo* colonization of metastatic cancer cells at healthy tissue sites in a patient and subsequent tumor growth^28^. Using this principle as demonstrated here, our technique can be used to construct patient-specific multifractal number libraries to aid in cancer disease progression in patients and follow disease remission after treatments are started.

## Discussion

Metastasis is a hallmark of cancer progression and accounts for most cancer-related deaths^28^. Higher metastatic potential resulting from higher motility due to increased fluidity of the cell membrane has been observed in well-established models of tumor progression^15–17^. Detecting these higher membrane hydration levels corresponding to higher metastatic potential by measuring the spectral properties of the cells^21^ has shown that ATR-FTIR spectroscopy is useful to distinguish different stages of cancer. To derive therapeutically relevant information, statistical evaluation of such acquired spectral data has been able to isolate spectral bands analogous to the increased levels of protein, lipid, and nucleic acid molecules in the serum of patients^29^. Thus, ATR-FTIR spectroscopy has become a remarkable tool in following cancer metastasis and prognosis. A method to combine such precise sensing of disease states into an objective digital output format can further enhance this technology to improve clinical practices in cancer treatment.

As discussed earlier, subjective pathological evaluation of biopsies remains the gold standard of cancer detection^4^ because of which successful clinical outcomes are limited by inter-observer and intra-observer discordance rates among pathologists^5, 6, 10^, specifically pertaining to how the medical information is processed, graded, and categorized. Our experiments demonstrate that the absorbance value of less malignant and more malignant cancer cells show a statistically significant difference, and we confirmed our hypothesis using a non-linear approach involving MFA of chaotic graphs that otherwise cannot be statistically quantified by a linear approach. By employing MFA on acquired FTIR spectra, we demonstrate that our technique makes it possible to objectively determine cancer metastatic potential directly correlated to their biochemical composition. Additionally, the technique is applicable to following disease progression and can potentially have application in monitoring disease remission after therapy.

A singularity spectrum describes the multifractal number of a subset of points of a function belonging to a group of points that have the same Holder exponent α (when a complex function *f* is distributed along *d* dimensional Euclidean space, then it satisfies the Holder exponent where Holder constant (α) is greater than 0). Intuitively, the singularity spectrum gives a value for how “fractal” a set of points are in a function. For a singularity spectrum, the probability of the distribution of space cannot be limited to just a set but should be studied using a more general framework. If any pattern shows non-uniformity that is associated with multifractals, then we consider *D*_*(Q)*_ versus *(Q)* where *D*_*(Q)*_ is generalized dimension and *Q* refers to arbitrary set of exponents, *f*_*(α)*_ versus *α* multifractal spectra where *f*_*(α)*_ is fractal dimension of set of boxes *α* (see *Methods*). In order to determine the random distribution of points, we characterize the distribution of data sets against the extent of distortion.

Till date, the standard intraoperative pathologic methods involving frozen section analysis and imprint cytology have been the traditional choices for intraoperative diagnosis during Breast Conserving Surgeries^30^. However, several issues remain in order for these techniques to generalize the method, such as the complexity and time-consuming nature of these procedures, as well as the demanding workload placed on pathologists. Our demonstrated technique may be a very practical solution to these problems. Our technique’s efficient processing and classifications can directly enhance and improve the clinical utility of a variety of other methods of surgical margin delimitation on live tissue, such as conventional specimen radiography (SR)^31^, intraoperative ultrasonography (IOUSG)^32^, radio-frequency spectroscopy (MarginProbe device)^33^, bioimpedance spectroscopy (ClearEdge device)^34^, microcomputed tomography (micro-CT)^35^, optical coherence tomography (OCT)^36^, ex vivo magnetic resonance imaging (ex vivo MRI)^37^, ultraviolet photoacoustic microscopy (UV-PAM)^38^, microscopy with ultraviolet surface excitation (MUSE)^39^ and other multimodal imaging techniques combining both macro (tissue-level) and micro (cell-level) detection capabilities.

The World Health Organization (WHO) has called for worldwide efforts to identify “best buys” and other cost-effective priority strategies for cancer prevention and control by developing precise standards and tools to guide the planning and implementation of interventions^1^. Recent years have seen the development of diagnostic tests based on attenuated total reflection (ATR)-Fourier transform infrared (FTIR) spectroscopy for the detection of cancer^40^ combined with machine learning technology^41^ demonstrating effectivity in triaging patients and allowing rapid access to appropriate treatment; although with limited throughput in most cases due to the requirement of developing specific instrumentations to undertake the novel signal processing methods. As such our innovative tool presented here combines the efficacy of fractal analysis matched to FTIR spectra processing to deliver a powerful diagnostic tool which can detect cancer cells at the most minute of concentrations as shown in our results above. Combined with the latest advances in endoscopic FTIR probes^21, 42, 43^ to complement conventional biopsies, our technique can transform the current levels of prevention, early diagnosis, screening, treatment, and palliative and survivorship care for both adult and child cancers by providing accurate technical assistance for rapid and effective transfer of best practice interventions to countries worldwide.

### Limitations

Multifractal analysis for spectra involves image processing, including choosing the number of pixels, grid size, and number of grids. Thus, multifractal analysis of FTIR spectra depends on the sampling procedure, type of sample and spectra results, and the establishment of different image processing parameters. MF analysis in FTIR involves no standard image processing protocol, and therefore loss of data from the spectra peaks may cause slight uncertainty in the results, which can be easily overcome by larger sampling numbers and studies involving various types of samples under different conditions to further improve the accuracy of the analysis and determine the best image processing protocol(s) for various applications.

The method demonstrated here uses a conversion of the ATR spectrum to a digital image, and the calculations are made on this binary image towards the conversion of information by the quantization of the spectrum. If the spectrum amplitude level is represented by 100 pixels (actually it is less than 100), the maximum signal to noise ratio that can be obtained is reduced to a value less than roughly 42 dB (6 times the number of bits, which is 7 for 128 pixels). Considering quantization of the wavenumber axis, it is be possible to obtain these methods based on the original spectrum levels. This article demonstrated the proof-of-concept encouraging the use of this novel method for preliminary approximate calculations currently, following which predictive testing on unknown samples will be subsequently investigated.

## Methods

### Cell lines

Mouse melanoma B16-f01 and B16-f10 cells, Human colorectal adenocarcinoma SW-480 and SW-620 cells and Normal murine fibroblast 3T3 cells were grown in growth medium composed of DMEM (Biological Industries, 01-050-1A, Beit Haemek, Israel)) supplemented with 10% FBS (04-007-1A, Biological Industries, Beit Haemek, Israel), 0.292 mg/mL L-glutamine (03-020-1B, Biological Industries, Beit Haemek, Israel) and 40 units/mL Penicillin–Streptomycin (03-031-1B Biological Industries, Beit Haemek, Israel).

### Cell suspension preparation

The cells were washed 1 × with PBS (02-023-1A Biological Industries, Beit Haemek, Israel) and trypsinised using Trypsin EDTA solution B (03-052-1B Biological Industries, Beit Haemek, Israel), and centrifuged at 400 g for 5 min at 4 °C and re-suspended in normal growth medium to the appropriate concentration as indicated in the experiments. 10 µl of sample was subsequently dropped on the diamond ATR in the FTIR instrument for acquiring absorbance spectra.

### ATR-FTIR spectroscopy

Measurements were carried out using an FTIR spectrometer (Jasco, 6800 FV, Tokyo, Japan) equipped with a Diamond ATR device (Jasco, ATR Pro One, Tokyo, Japan). The effective dimensions of the diamond are 1.8 mm diameter. The refractive index of the diamond is 2.4 and the angle of incidence in our device was 45°, generating 1 reflection. The calculated depth of penetration is ~ 2 μm. The radiation from the IR source of the spectrometer was focused into the ATR diamond, and the output radiation (from the other side of the diamond) was focused onto a DLaTGS (Deuterated Lanthanum α Alanine doped TriGlycine Sulphate) detector. 10–20 µl of cell suspensions of specific lineages were placed on the diamond ATR. Pressure (700 kg/cm^2^) was applied to produce better contact between the sample and the diamond. Measurements were carried out in the spectral range of 4000–650 cm^−1^. Each spectrum at acquisition (see *Supplementary*) was an average of 64 scans to increase the signal to noise ratio (SNR). The spectra were analyzed using Spectra Analysis™ (Jasco, Tokyo, Japan).

### Multifractal analysis

The Multifractal spectrum is characterized by a continuum of singularities, which is defined by α, and the fractal dimension f_(α)_ defined by their sets. In order to determine the random distribution of points, we characterized the distribution of data sets against the size of the distorting lens. Unlike fractal dimension where f = α = D where D is fractal dimension, multifractal dimension is obtained by calculating generalized dimension D_(Q)_, singularity spectrum f_(α)_ and fractal dimension D. Considering all the distribution of data sets, multifractal analysis gives a more accurate value concerning any given property of self-similarity.

Multifractal analysis is based on the box counting method, where each pixel in a digital image contains a distribution of sets. To compare and quantify various features of a multifractal spectra, we used three data sets, such as “Dimensional ordering”, “Singularity spectrum *f*_(α)_” and “Fractal dimension” D.

#### Generalized dimension

A generalized dimension or D_(Q)_, is defined by the distortion of the mean (µ) for a distribution of pixel values at a certain level of complexity ε resulting from box counting. To calculate the generalized dimension D_(Q)_, µ is raised to the arbitrary exponent Q and then compared to how this rise varies to ε which determines how mass varies with ε (resolution/box size).

**Figure 3:**
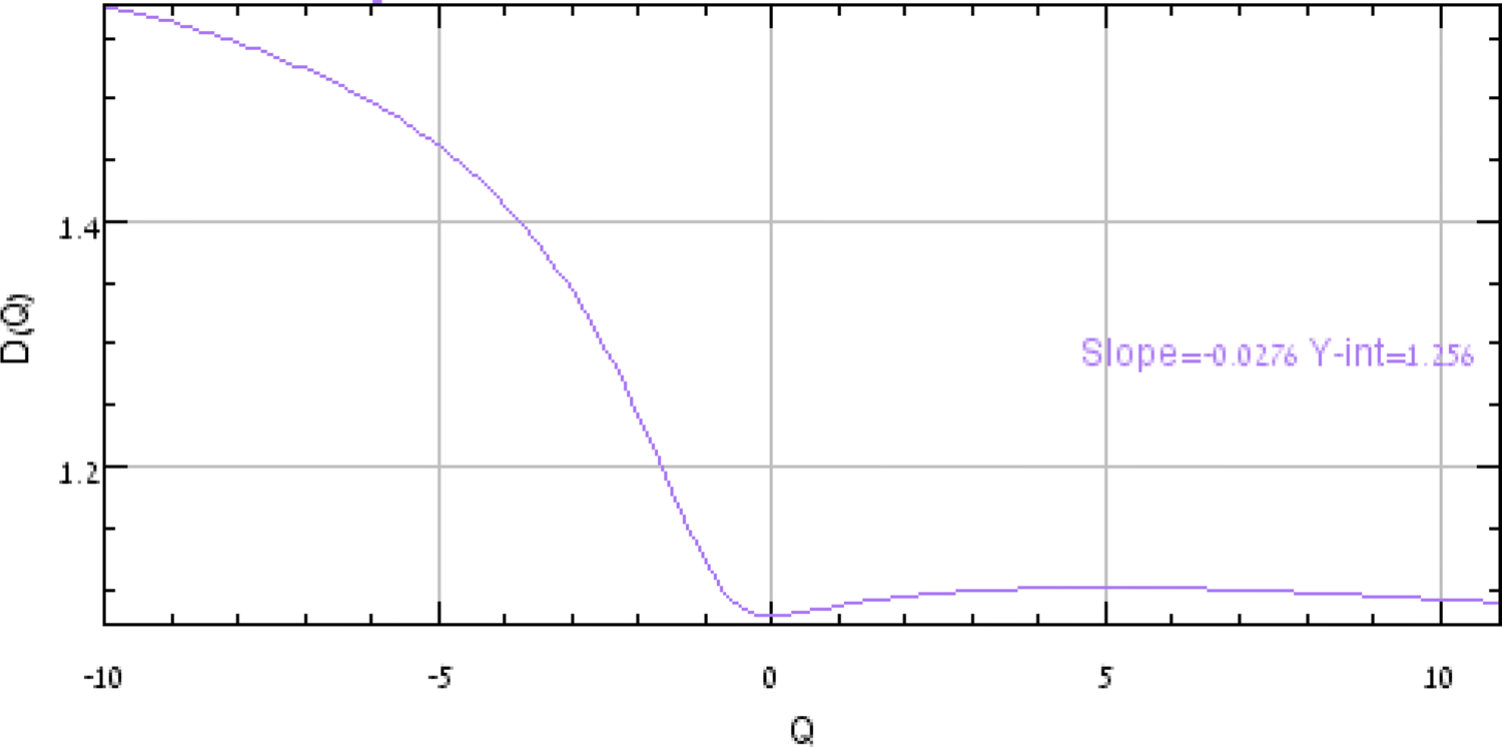
D(Q) versus Q (0) for a multifractal spectra.

The graphical representation of D_(Q)_ versus Q aids in distinguishing between different types of patterns (Fig. 3), giving us a sigmoidal curve around Q = 0 and the pattern varies between non-fractals (a geometrical figure that is self-similar in all scales monofractals) and multifractals (a generalized system of fractals where a data is more complex to be described only by fractal dimension) as shown below, where,

$${\text{D}}_{{({\text{Q}})}} = \frac{{\tau \left( {\text{Q}} \right)}}{{{\text{Q}} - 1}}$$

$${\text{D}}_{{({\text{Q}}\; = \;0)}} \ge {\text{ D}}_{{({\text{Q}}\; = \;1)}} \ge {\text{ D}}_{{({\text{Q}}\; = \;2)}}$$

For a multifractal data, the typical *f*_*(α)*_ versus *α* shows humped spectra whereas in case of mono-fractals they converge at a certain value (Fig. 3).

In order to determine differences between two samples using an FTIR absorbance spectra, MFA plays an interesting role in statistically predicting and distinguishing the abnormalities in the two samples. Therefore, to determine if the current spectrum is a multifractal, we need to analyze the singularity spectrum.

#### Singularity spectrum

**Figure 4:**
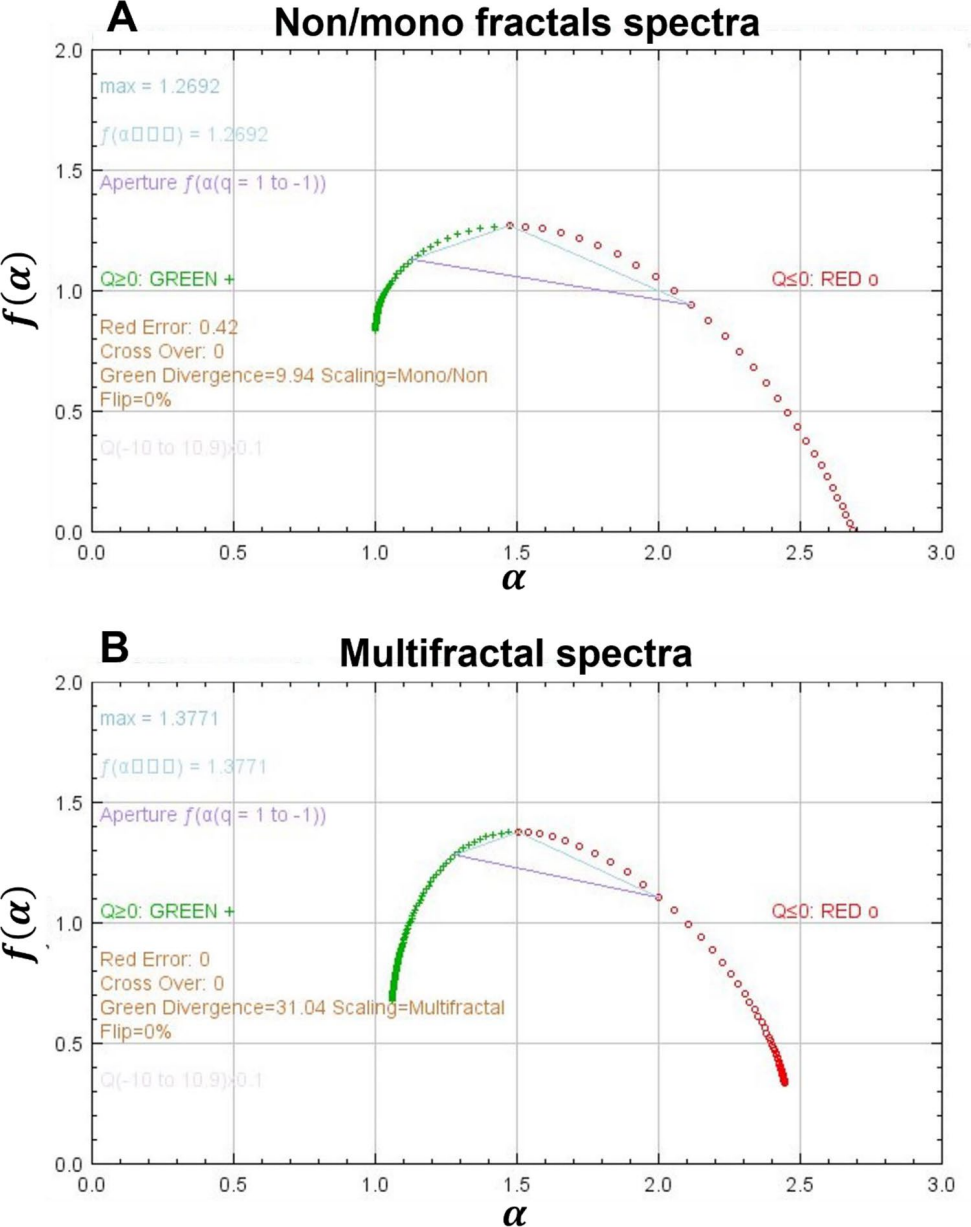
(**A**) Singularity spectrum of non/mono fractals (**B**) Singularity spectrum of multifractals showing a typical hump. The α_(min)_ (green dots) and α_(max)_ (Red dots) reveals how strong the multifractal is in the given subject.

To quantitate the multifractal spectrum or singularity spectrum, we evaluate the aperture, which is determined by an intersection between *f*_(α)_ and α between positive and negative values, Q = 1 and Q = − 1, and from the lines where Q = 0. Non, mono and multifractals can be distinguished using a standard *f*_(α)_ versus α graph (Fig. 4).

The singularity spectrum *f*_*α*_ can be determined by τ_(Q):_

$$\tau_{{({\text{Q}})}} = {\text{ Q X H}}_{{({\text{Q}})}} {-} \, 128$$

$$\alpha \, = \frac{{\partial \tau \left( {\text{Q}} \right)}}{{\partial {\text{Q}}}}$$

### Image processing

**Figure 5:**
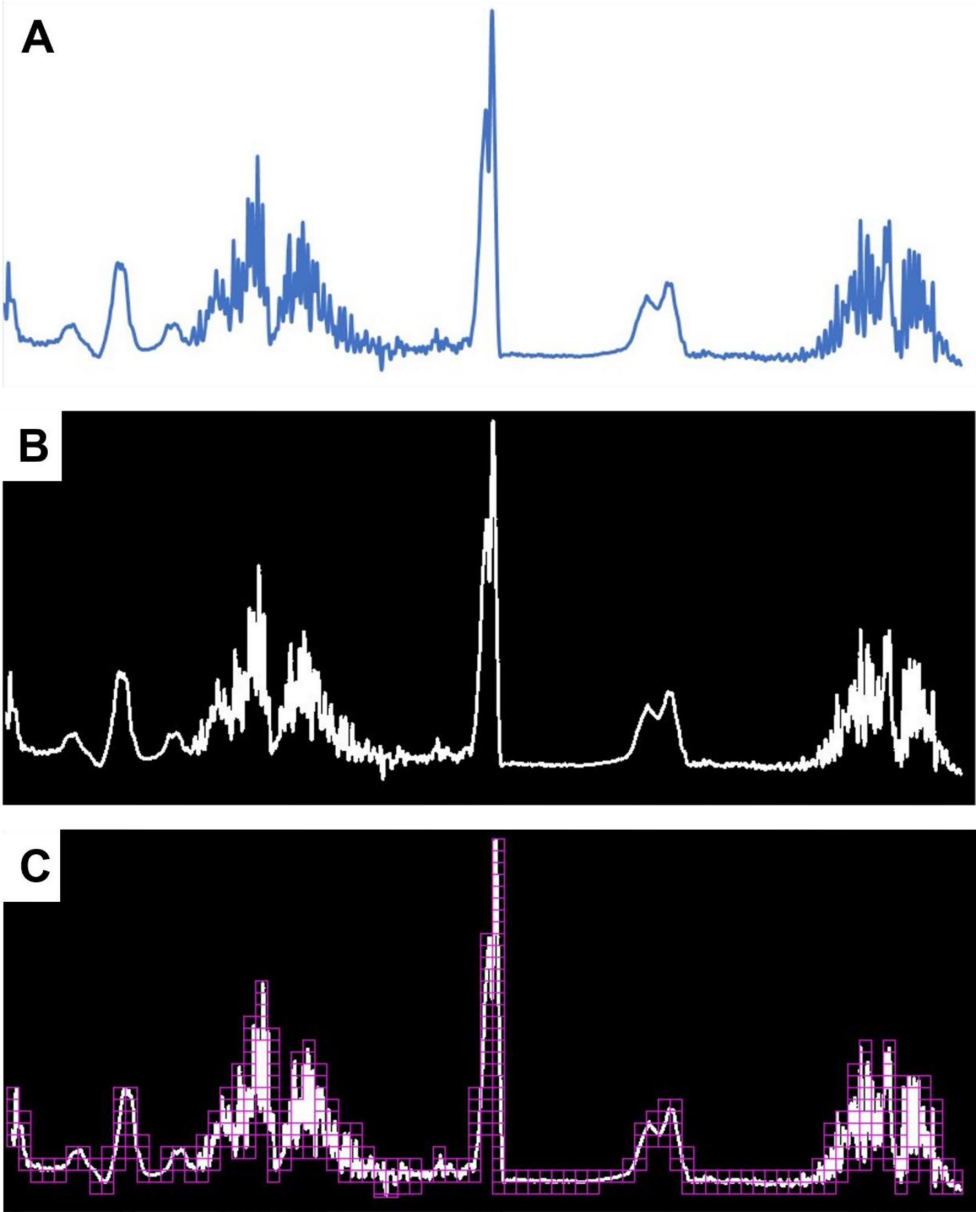
Image processing protocol. (**A**) Raw data of ATR-FTIR spectra eliminating grids and axis for MF analysis. (**B**) 8-bit grey scale image of ATR-FTIR spectra compatible for MF analysis. (**C**) Number of grids covering ATR-FTIR spectra to determine number of pixels covered by each grid.

ATR-FTIR was used to examine the mid-infrared absorbance spectra (4000–650 cm^−1^) of murine melanoma cell lines with low and high metastatic potential (B16-f01 vs. B16-f10) and lower and higher metastatic potential colorectal cancer (SW-480 vs. SW-620). spectra were processed in BMP format followed by converting them into binary images, which were then analyzed for fractal dimension (mono-fractal) and multifractal by setting box/grid sizes to 100, minimum pixel size to 10, and maximum image percentage to 60 (Fig. 5). The acquired spectra were analyzed using FracLac plugin (Karperien, A., FracLac for ImageJ. http://rsb.info.nih.gov/ij/plugins/fraclac/FLHelp/Introduction.htm. 1999–2013) in ImageJ software (Rasband, W.S., ImageJ, U. S. National Institutes of Health, Bethesda, Maryland, USA, https://imagej.nih.gov/ij/, 1997–2018). The Q value was set between − 10 and 10, and graphs were plotted for generalized dimension D_(Q)_ and singularity spectrum f_(α)_.

**Figure 6:**
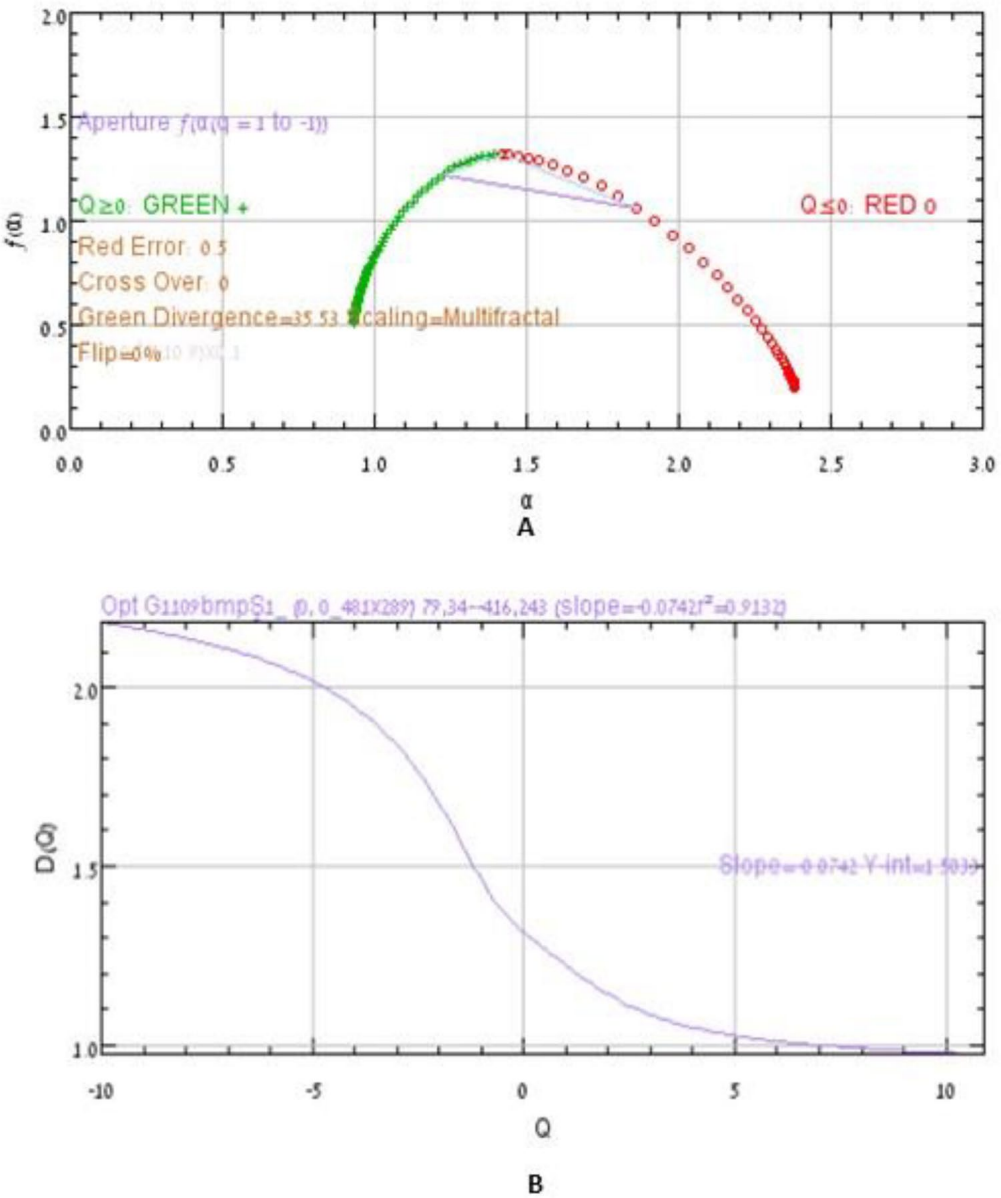
Graphical representation of f(α) versus α (**A**) and D(Q) versus Q (**B**) plot for the given spectrum.

The D_(Q)_ versus Q and *f*_(α)_ versus α graphs both showed typical multifractal behavior, thus confirming that the spectrum analysed was not a simple monofractal in fact a multifractal structure (Fig. 6).

### Data processing

Statistical analysis and graphical representation were done using GraphPad prism software (GraphPad Prism version 8.0.0 for Windows, GraphPad Software, San Diego, California USA, www.graphpad.com).

### Supplementary Information

Supplementary Figures.

## Data Availability

All data are available from authors upon request.
