# Supplementary material for: Multifractal analysis of cellular ATR-FTIR spectrum as a method for identifying and quantifying cancer cell metastatic levels
Source: Sci Rep. 2023 Nov 2;13:18935. doi: 10.1038/s41598-023-46014-1 (PMC10622493; doi:10.1038/s41598-023-46014-1)
Supplement: Supplementary file 1 — Supplementary Figures. [file 41598_2023_46014_MOESM1_ESM.docx]

**Multifractal analysis of cellular ATR-FTIR spectrum as a method for identifying and quantifying cancer cell metastatic levels.**

Ayan Barbora^1,+^, Sirish Karri^2,+^, Michael A Firer^2,3,4^, Refael Minnes^1,*^

^1^ Department of Physics, Ariel University, Ariel, 40700, Israel

^2^ Department Chemical Engineering, Ariel University, Ariel, 40700, Israel

^3^ Adelson School of Medicine, Ariel University, Ariel 40700, Israel

^4^ Ariel Center for Applied Cancer Research, Ariel University, Ariel 40700, Israel

[^*^ refaelm@ariel.ac.il](mailto:*corresponding.author@email.com)

^+^these authors contributed equally to this work


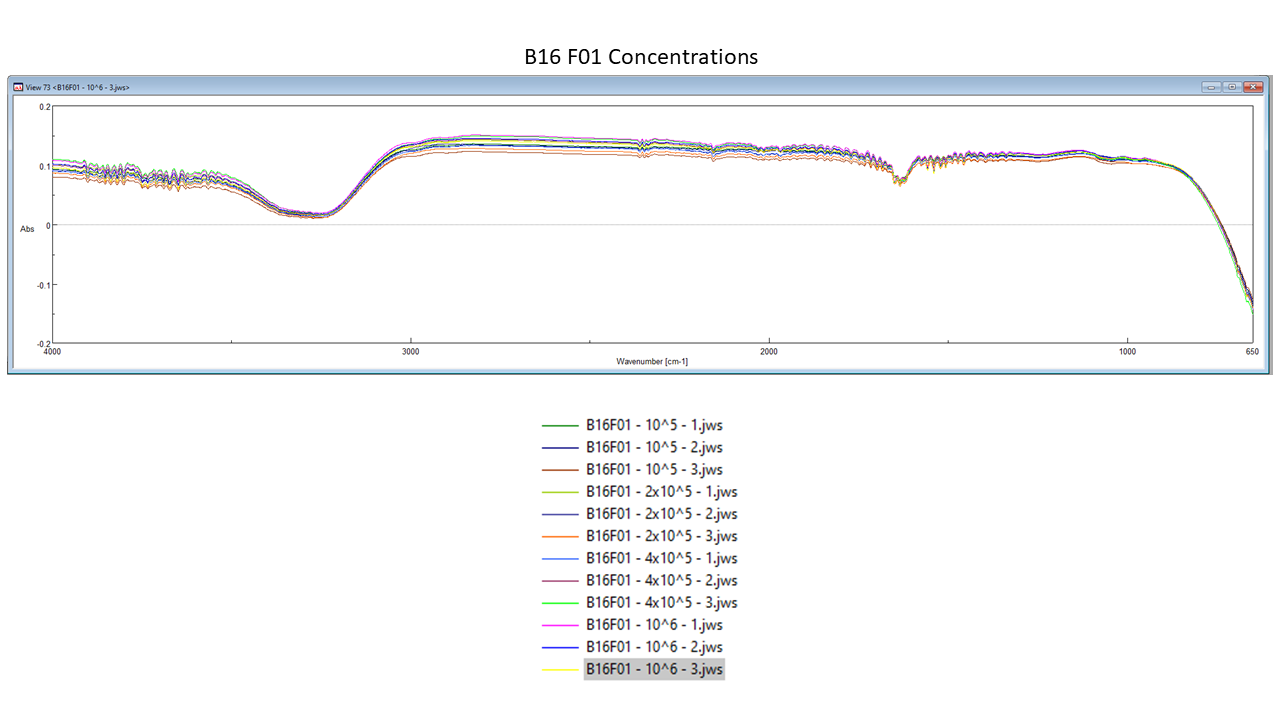


**Figure S1**. ATR-FTIR Spectra from B16-F01 cell lines at subsequently increasing concentration levels (10^5^, 2x10^5^, 4x10^5^ and 10^6^ cells/ml). Measurements involved ATR-FTIR spectra from 3 distinct samples for each respective concentration as indicated.


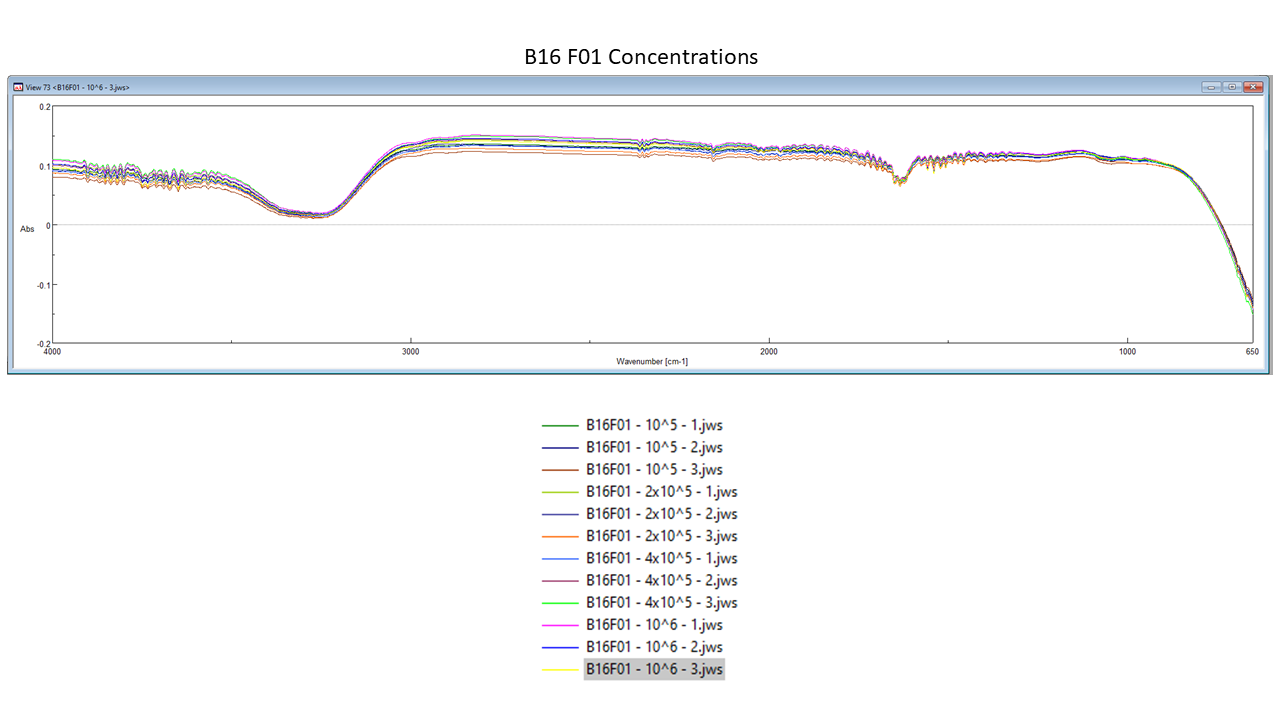


**Figure S2**. ATR-FTIR Spectra from B16-F10 cell lines at subsequently increasing concentration levels (10^5^, 2x10^5^, 4x10^5^ and 10^6^ cells/ml). Measurements involved ATR-FTIR spectra from 3 distinct samples for each respective concentration as indicated.


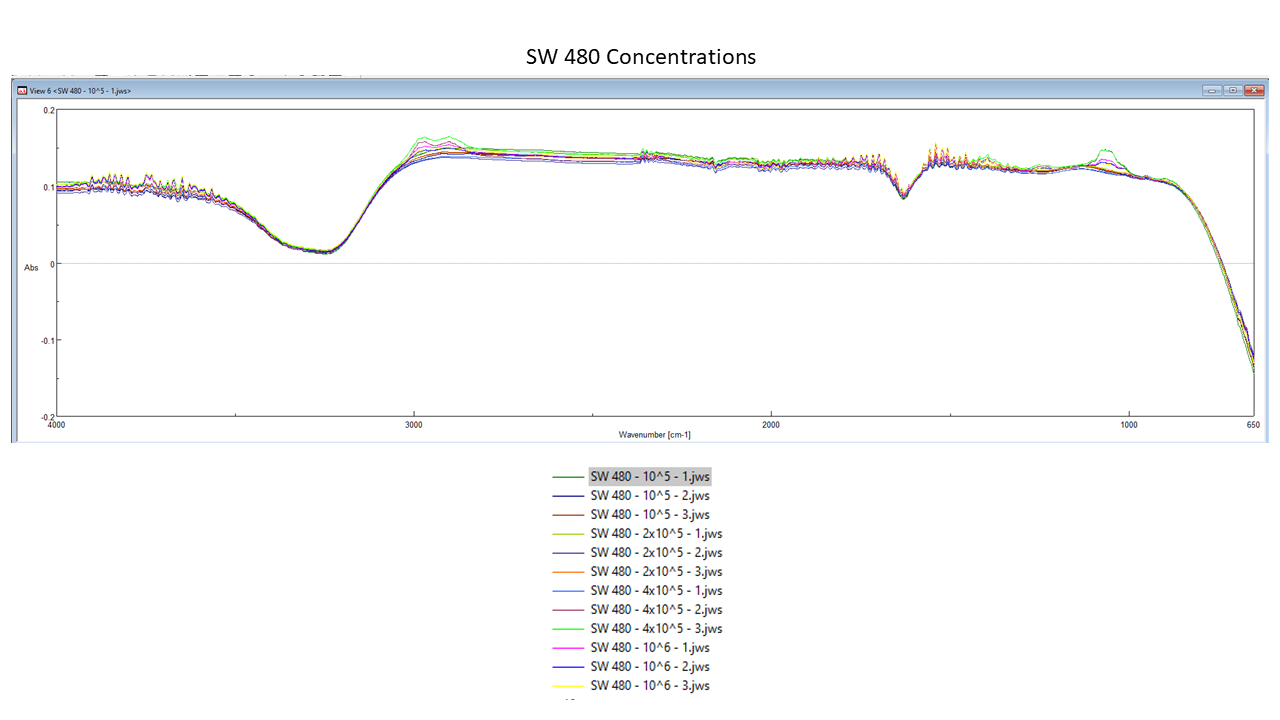


**Figure S3**. ATR-FTIR Spectra from SW-480 cell lines at subsequently increasing concentration levels (10^5^, 2x10^5^, 4x10^5^ and 10^6^ cells/ml). Measurements involved ATR-FTIR spectra from 3 distinct samples for each respective concentration as indicated.


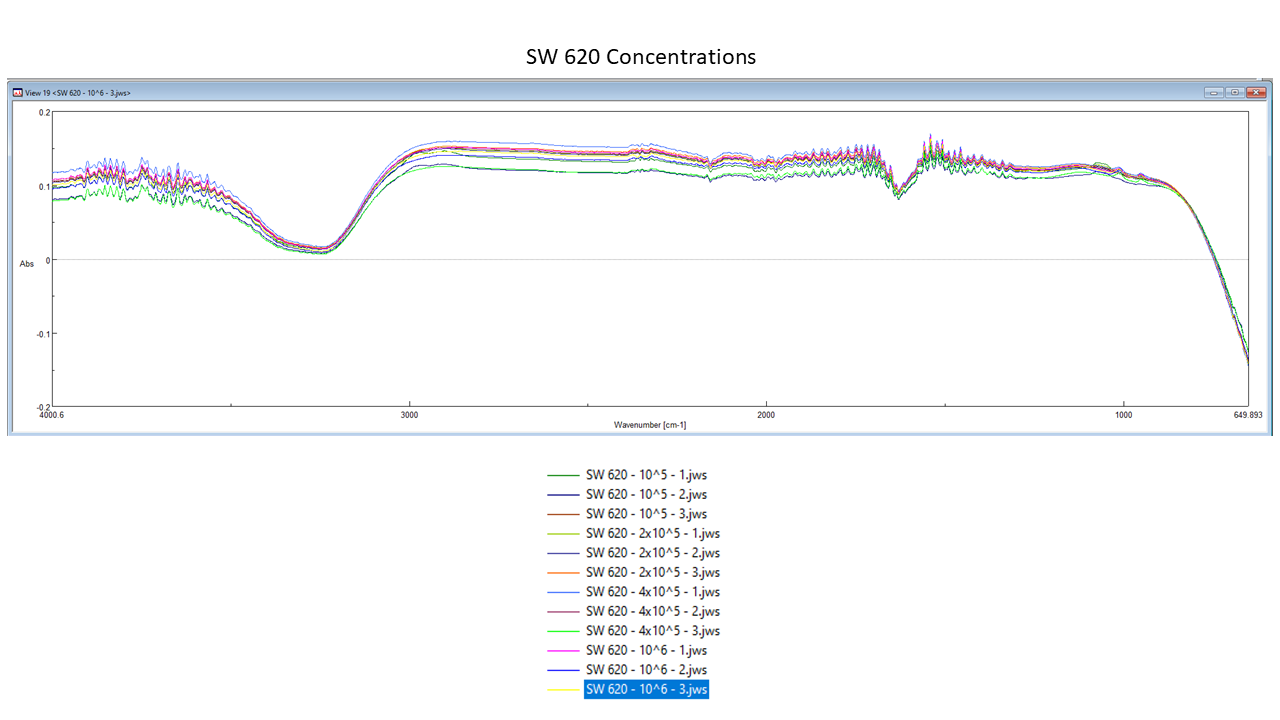


**Figure S4**. ATR-FTIR Spectra from SW-620 cell lines at subsequently increasing concentration levels (10^5^, 2x10^5^, 4x10^5^ and 10^6^ cells/ml). Measurements involved ATR-FTIR spectra from 3 distinct samples for each respective concentration as indicated.


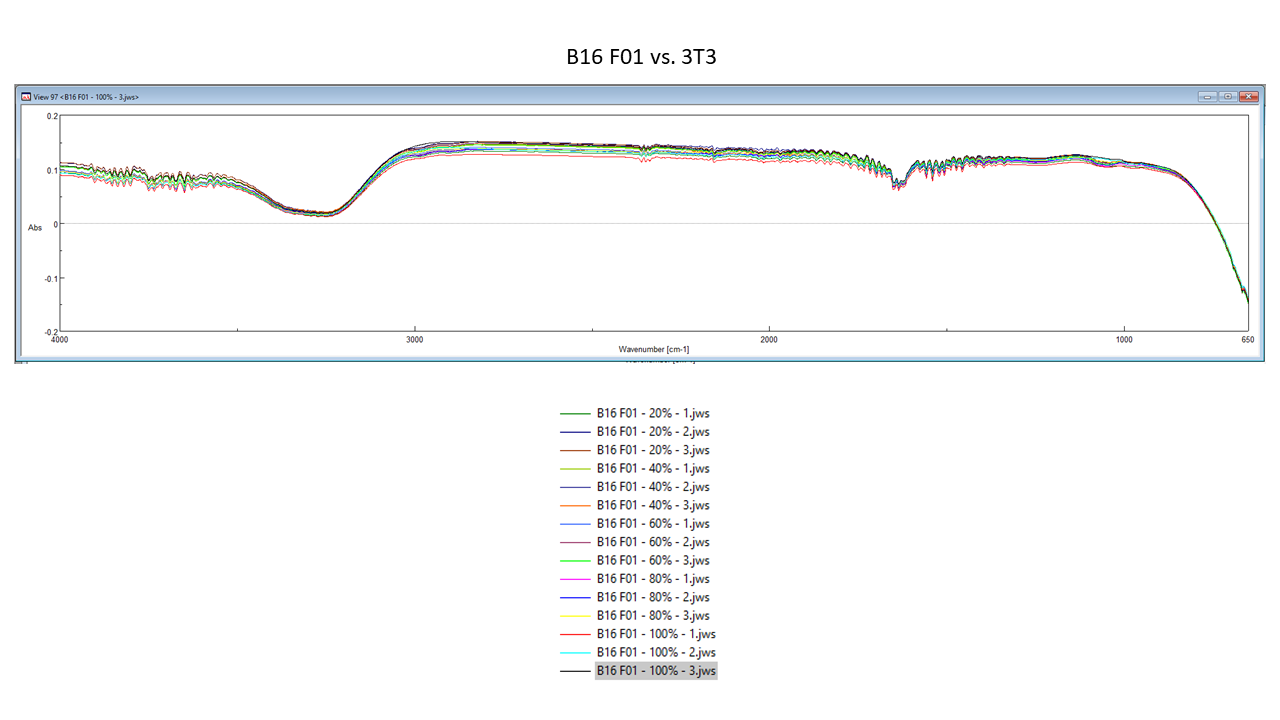


**Figure S5**. ATR-FTIR Spectra from B16-F01 cells mixed with 3T3 cells at various percentages. Measurements involved ATR-FTIR spectra from 3 distinct samples for each respective concentration as indicated.


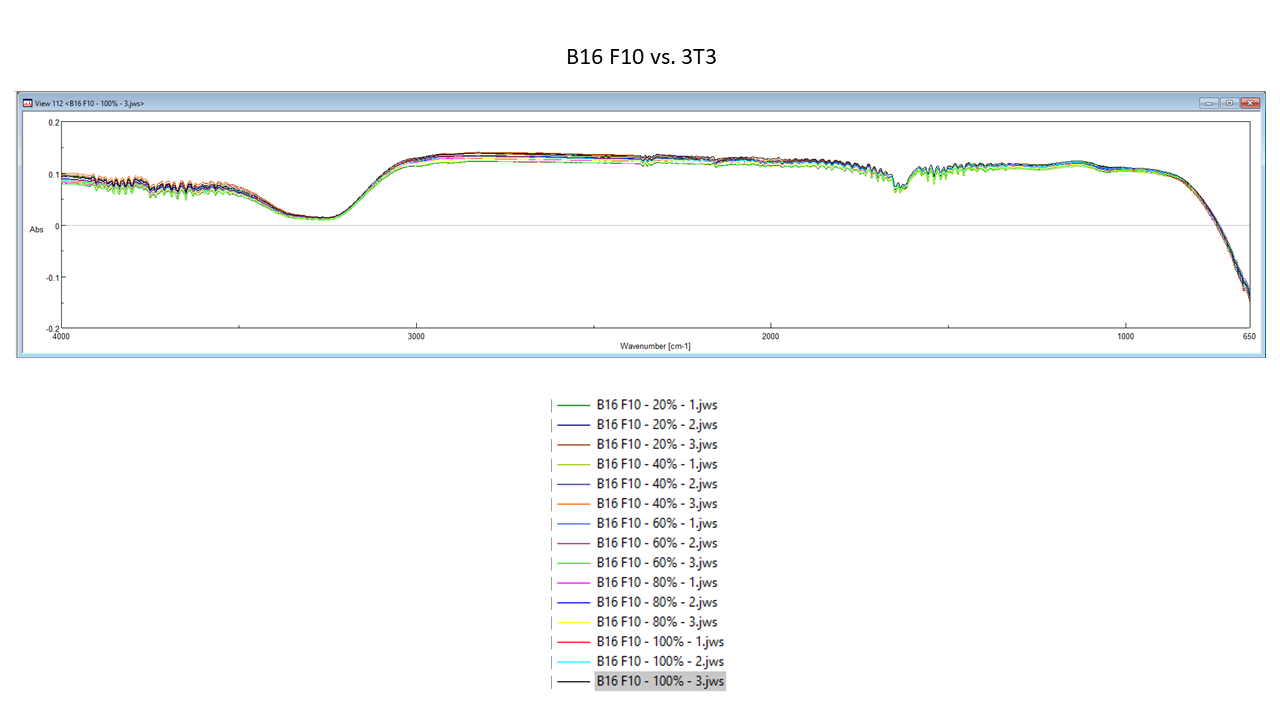


**Figure S6**. ATR-FTIR Spectra from B16-F10 cells mixed with 3T3 cells at various percentages. Measurements involved ATR-FTIR spectra from 3 distinct samples for each respective concentration as indicated.


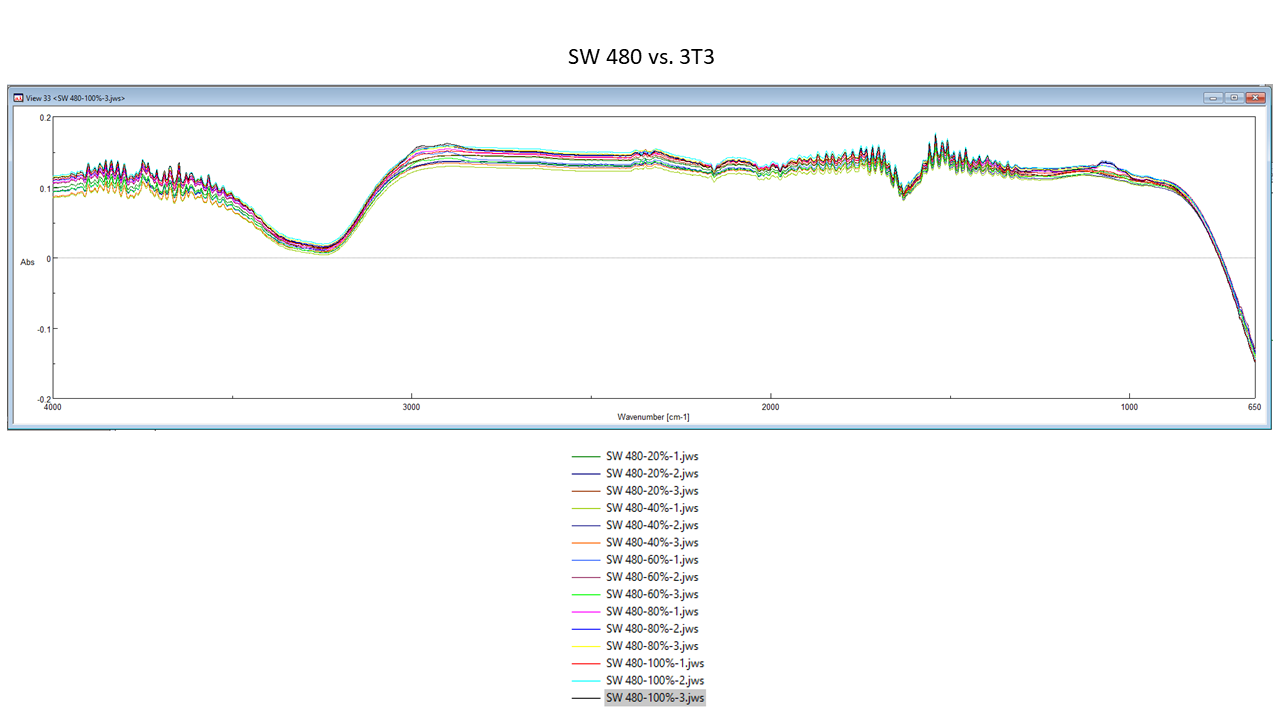


**Figure S7**. ATR-FTIR Spectra from SW-480 cells mixed with 3T3 cells at various percentages. Measurements involved ATR-FTIR spectra from 3 distinct samples for each respective concentration as indicated.


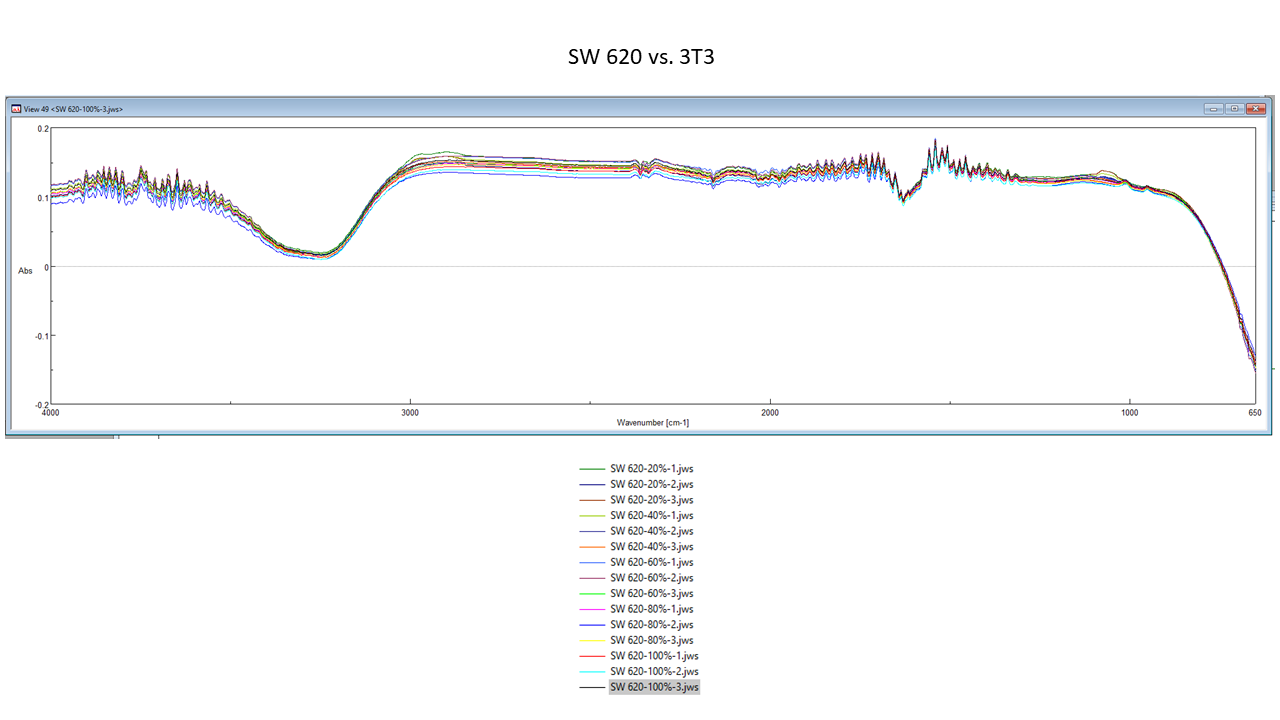


**Figure S8**. ATR-FTIR Spectra from SW-620 cells mixed with 3T3 cells at various percentages. Measurements involved ATR-FTIR spectra from 3 distinct samples for each respective concentration as indicated.


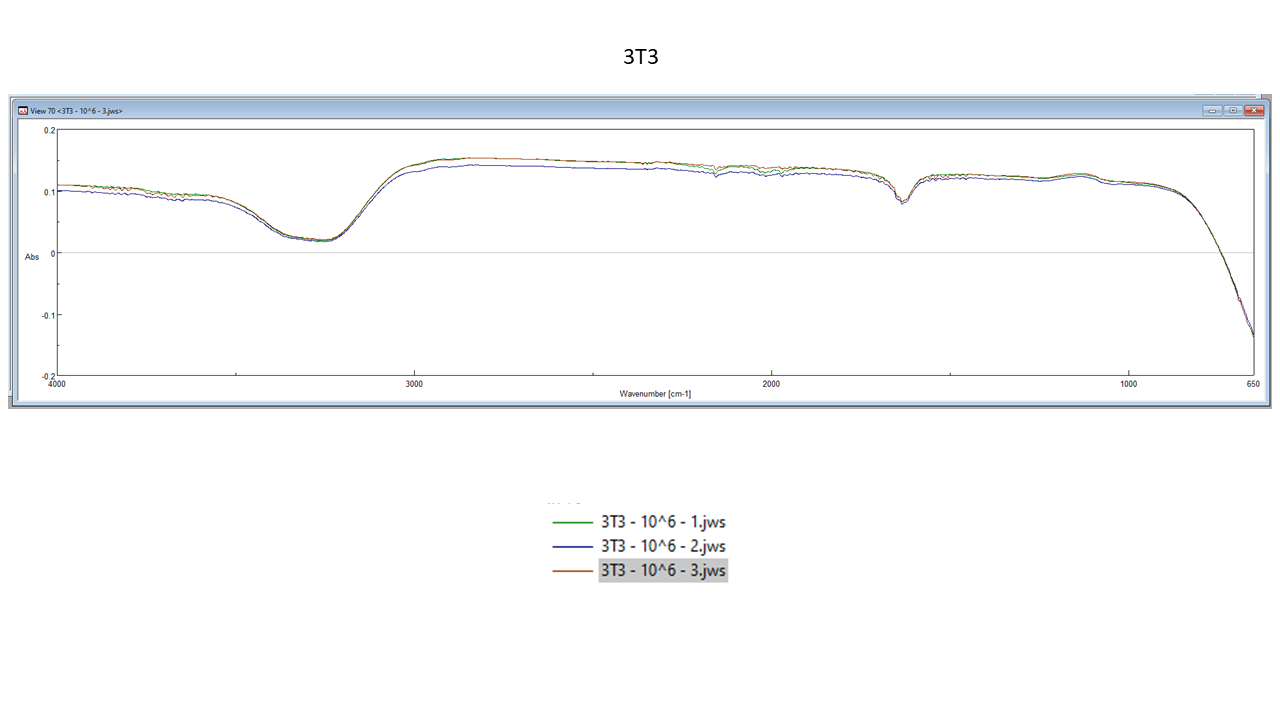


**Figure S9**. ATR-FTIR Spectra from 3T3 cells. Measurements involved ATR-FTIR spectra from 3 distinct samples.
